# Three-Dimensional Bowing Measurement of Distal Femur at Actual Size and Clinical Implications of Total Knee Arthroplasty

**DOI:** 10.3390/medicina60060986

**Published:** 2024-06-15

**Authors:** Gu-Hee Jung, Young-Jue An, Dong-Geun Kang

**Affiliations:** Department of Orthopaedic Surgery, College of Medicine, Gyeongsang National University Changwon Hospital, 11 Samjungja-ro, Changwon-si 51472, Republic of Korea; jyujin2001@hotmail.com (G.-H.J.); theenvy5@gmail.com (Y.-J.A.)

**Keywords:** Asian femur, three-dimensional model, computational simulation, total knee arthroplasty, cortical abutment

## Abstract

*Background and Objectives*: To assess femoral shaft bowing (FSB) in coronal and sagittal planes and introduce the clinical implications of total knee arthroplasty (TKA) by analyzing a three-dimensional (3D) model with virtual implantation of the femoral component. *Materials and Methods*: Sixty-eight patients (average age: 69.1 years) underwent 3D model reconstruction of medullary canals using computed tomography (CT) data imported into Mimics^®^ software (version 21.0). A mechanical axis (MA) line was drawn from the midportion of the femoral head to the center of the intercondylar notch. Proximal/distal straight centerlines (length, 60 mm; diameter, 1 mm) were placed in the medullary canal’s center. Acute angles between these centerlines were measured to assess lateral and anterior bowing. The acute angle between the distal centerline and MA line was measured for distal coronal and sagittal alignment in both anteroposterior (AP) and lateral views. The diameter of curve (DOC) along the posterior border of the medulla was measured. *Results*: The mean lateral bowing in the AP view was 3.71°, and the mean anterior bowing in the lateral view was 11.82°. The average DOC of the medullary canal was 1501.68 mm. The average distal coronal alignment of all femurs was 6.40°, while the distal sagittal alignment was 2.66°. Overall, 22 femurs had coronal bowing, 42 had sagittal bowing, and 15 had both. *Conclusions*: In Asian populations, FSB can occur in coronal, sagittal, or both planes. Increased anterolateral FSB may lead to cortical abutment in the sagittal plane, despite limited space in the coronal plane. During TKA, distal coronal alignment guides the distal femoral valgus cut angle, whereas distal sagittal alignment aids in predicting femoral component positioning to avoid anterior notching. However, osteotomies along the anterior cortical bone intended to prevent notching may result in outliers due to differences between the distal sagittal alignment and the distal anterior cortical axis.

## 1. Introduction

When performing total knee arthroplasty (TKA) as the favored intervention for severe osteoarthritis, achieving proper lower limb alignment and optimal implant positioning is imperative to attain favorable outcomes. To address these concerns, along with the surgeon’s proficiency in relevant surgical techniques, preoperative assessment of the femur shaft’s morphology in both coronal and sagittal planes is crucial [1,2]. The impact of femoral shaft bowing (FSB) on implant positioning in TKA has garnered increasing attention, particularly among Asian patients with osteoarthritis [1]. Although a precise definition and measurement method for FSB is lacking [3,4], prior research suggests its racial specificity, notably affecting Asians to a greater extent [1,5].

Recent studies on atypical fractures [4,6,7] and computational anatomy [8,9,10] have presented bowing measurement methods utilizing plain radiographs as the basis. However, these methods are prone to projection errors during preoperative planning and encounter difficulties in synchronizing the alignment of the medullary canal centerline in both coronal and sagittal planes. Based on clinical observations, we recognized the necessity for a detailed understanding of FSB in these planes, utilizing an actual-sized Asian femur model. Consequently, this study developed a clinical computed tomography (CT)-based Asian femur model to assess three-dimensional (3D) FSB, establishing a synchronized centerline through axial, coronal, sagittal, and 3D biplanar images [9].

The main objectives of this clinical CT-based study were to assess FSB in the coronal and sagittal planes without projection error and to compare it with the femoral mechanical axis (MA) in the Asian ethnicity. Additionally, this study aimed to evaluate the morphological features of the medullary canal as a potential space for intramedullary (IM)-guided rods and implants. Finally, it sought to demonstrate the practical implications of TKA by analyzing a 3D model with virtual implantation of the femoral component and stem.

## 2. Materials and Methods

**Figure 1:**
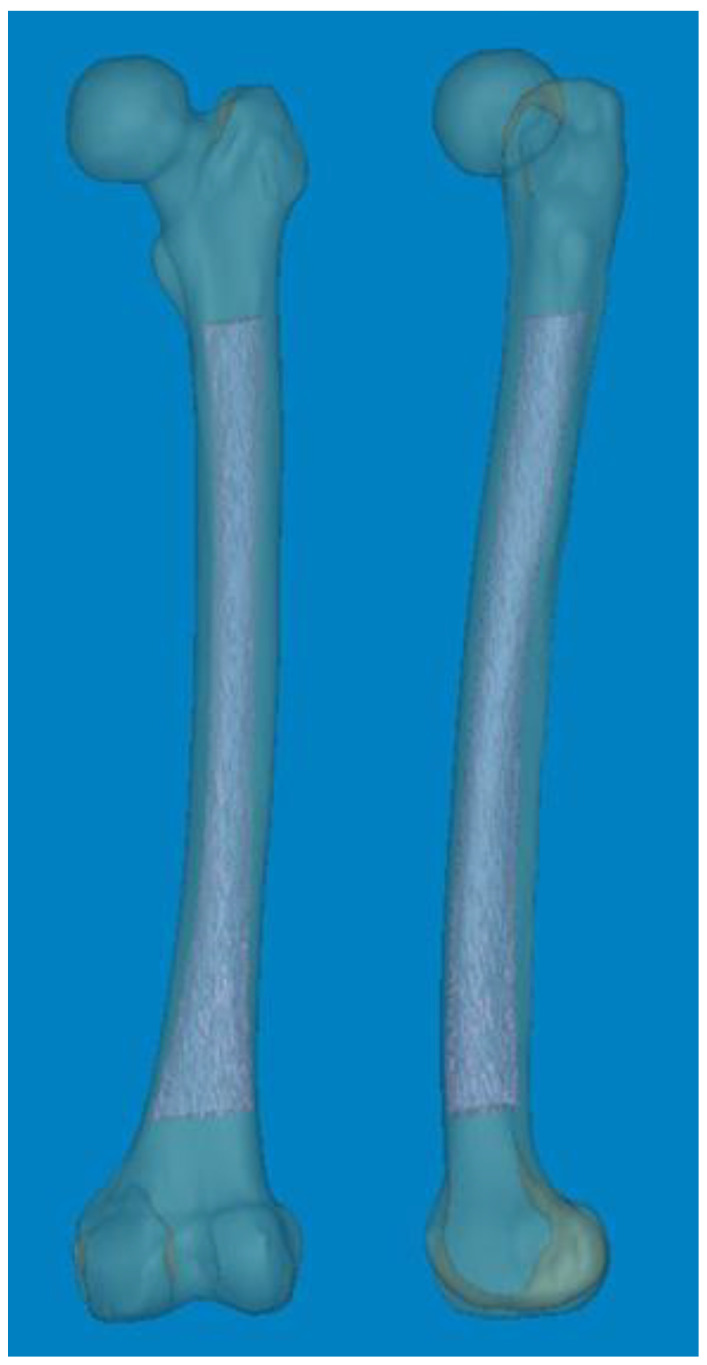
Mimics^®^ software (version 21.0) was used to reconstruct 3D femur models, including the medullary canal at actual size.

We conducted a retrospective analysis of medical records and radiographs of non-injured femurs. The initial pool comprised patients who had undergone CT scan of both femurs following fractures. Patients with metastatic bone tumors, hip and knee joint deformities, and a history of fracture surgery in the ipsilateral limb were subsequently excluded after reviewing medical history and radiographs. Ultimately, 68 patients (35 males and 33 females) with an average age of 69.1 years (range: 33–93 years; standard deviation [SD]: 13.97) were enrolled. CT data in the Digital Imaging and Communications in Medicine format were imported into Mimics^®^ software (Materialise Interactive Medical Image Control System; Materialise, Antwerp, Belgium). Non-injured femurs were selected as the region of interest for reconstructing Asian femur models, with other anatomical structures such as the pelvis and leg being removed in order to streamline 3D rendering. Subsequently, the entire femurs were anatomically realigned to resemble a standing position (Figure 1).

**Figure 2:**
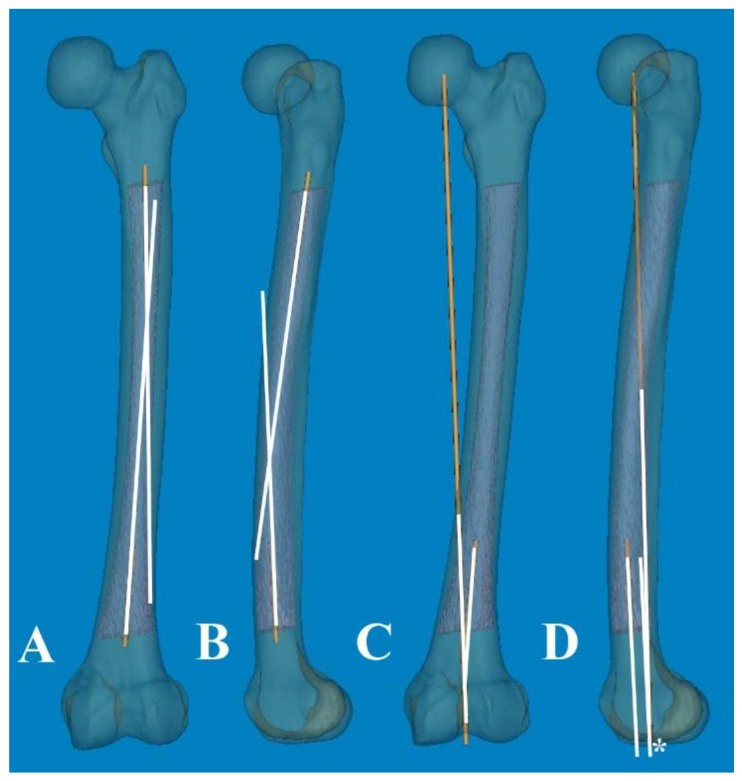
(**A**,**B**) An acute angle formed by the meeting of two straight centerlines drawn was considered lateral and anterior bowing. (**C**,**D**) The acute angle by the straight centerline and mechanical axis line was measured as distal coronal and sagittal alignment. Color; 3D reconstruction image of the Medullary canal. *; center of intercondylar notch of femur.

**Figure 3:**
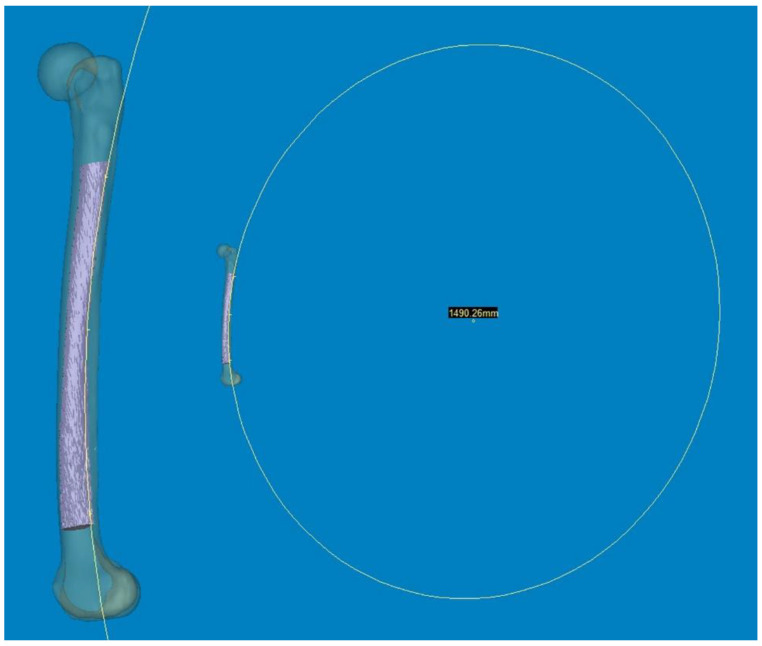
Three points along the posterior surface of the medullary canal were selected. The first point was located 2 cm below the lesser trochanter. The second point was located 2 cm above the distal metaphyseal flare. The third point was equidistant between the two. According to these three points, the diameter of the curve (DOC) of the medullary canal along the posterior border was measured.

To measure 3D FSB and MA at the actual size, the Mimics^®^ software was employed with the following steps [11,12]: (1) A mechanical line was virtually inserted and aligned from the center of the femoral head to the center of the intercondylar notch. (2) Two straight centerlines (60 and 1 mm in length and diameter, respectively) that bisected the transverse diameter of the proximal and distal shafts were virtually inserted in the proximal/distal end of the shaft and placed centrally in any direction from the outer boundary of the medullary canal. The acute angle formed by the meeting of the two lines was then considered diaphyseal bowing in the coronal plane (lateral bowing) and sagittal plane (anterior bowing) [7]. (3) The acute angle between the distal centerline and the mechanical line was measured to determine the distal coronal and sagittal alignment (Figure 2) [7]. (4) The measurement tools within the Mimics^®^ software were used to provide a reliable selection of points. Three points along the posterior surface of the medullary canal were marked (point A was located 2 cm below the lesser trochanter, point C was located 2 cm above the distal metaphyseal flare, and point B was equidistant between points A and C). According to these three points, the diameter of curve (DOC) along the posterior border of the medullary canal was measured (Figure 3). Finally, experienced surgeons fine-tuned and verified the definitive positions of all measurement tools.

All patients were of Korean ethnicity, with 40 aged over 70 years. This study was divided into four age groups: Group I (under 60 years old) consisted of 14 participants; Group II (60 to 69 years old) also had 14 participants; Group III (70 to 79 years old) included 21 participants, and Group IV (over 80 years old) comprised 19 participants. FSB > 5° in the coronal plane was classified as lateral bowing (+), whereas FSB of >11° in the sagittal plane was categorized as anterior bowing (+) [3].

All measurements are presented as means with standard deviations (SDs) or medians with interquartile ranges (IQRs), depending on the distribution of the data. Continuous variables were assessed for normality using the Shapiro–Wilk test. Normally distributed data are expressed as mean ± SD, while non-normally distributed data are expressed as median (IQR). Categorical variables are presented as frequencies and percentages. To compare continuous variables between two groups, the independent samples t-test was used for normally distributed data, and the Mann–Whitney U test was used for non-normally dis-tributed data. For comparisons among four groups, ANOVA was employed, followed by Scheffe’s post hoc test for multiple comparisons. Chi-square tests were used to compare categorical variables. Correlation analysis was performed using Pearson’s correlation coefficients for normally distributed data and Spearman rank correlation coefficients for non-normally distributed data. Statistical analyses were performed using the SPSS statistical software package for Windows version 25.0 (SPSS Inc., Chicago, IL, USA). A value of *p* < 0.05 was considered to be statistically significant. 

## 3. Results

The summary of femur characteristics are presented in Table 1. Concerning FSB, the average lateral bowing in the anteroposterior (AP) view was 3.71° (range: 0.0–10.1°; SD: 2.71), and the average anterior bowing in the lateral view was 11.82° (range: 4.5–18.5°; SD: 2.97). The mean DOC of the medullary canal was 1501.68 mm (range: 979.6–2515.8 mm; SD: 317.70). The distal coronal alignment averaged 6.40° (range: 1.98–11.17°; SD: 2.14), and the distal sagittal alignment averaged 2.66° (range: −1.24–7.35°; SD: 2.06). Coronal FSB was observed in 22 cases, sagittal FSB in 42 cases, and 15 femurs exhibited both types. Analyzing by sex, males had a lateral bowing of 2.37 ± 2.05°, anterior bowing of 11.17 ± 2.62°, distal coronal alignment of 5.61 ± 1.84°, distal sagittal alignment of 2.16 ± 1.60°, and DOC of 1567.11 ± 339.41 mm. Conversely, females showed values of 5.14 ± 2.63° for lateral bowing, 12.50 ± 3.20° for anterior bowing, 7.24 ± 2.16° for distal coronal alignment, 3.18 ± 2.38° for distal sagittal alignment, and a DOC of 1432.29 ± 281.56 mm. Comparisons between the two groups revealed statistically significant differences in lateral bowing, distal coronal alignment, and sagittal alignment (*p* < 0.05). Pearson’s chi-square test for sex difference indicated *p*-values of 0.006 for coronal FSB (22 femurs) and 0.419 for sagittal FSB (42 femurs).

The results based on an age cutoff of 70 years are detailed in Table 2. Participants younger than 70 years exhibited lateral bowing values of 2.02 ± 1.60°, anterior bowing of 10.60 ± 2.50°, distal coronal alignment of 5.22 ± 1.51°, distal sagittal alignment of 1.90 ± 1.66°, and a medullary canal diameter of 1601.81 ± 364.95 mm. Participants older than 70 years showed values of 4.90 ± 2.72° for lateral bowing, 12.67 ± 3.01° for anterior bowing, 7.23 ± 2.15° for distal coronal alignment, 3.19 ± 2.17° for distal sagittal alignment, and a medullary canal diameter of 1431.59 ± 262.58 mm. These differences were statistically significant (*p* < 0.05). Regarding those aged 70 years, Pearson’s chi-square test showed *p*-values of 0.001, 0.027, and 0.000 for coronal FSB, sagittal FSB, and both FSB, respectively. A summary of the four groups is presented in Table 3. Analysis of variance comparing the means of the four groups revealed homogeneity of variances (*p* > 0.05). Analysis of variance tests for lateral bowing, anterior bowing, distal coronal alignment, and DOC were significant (*p* < 0.005), whereas distal sagittal alignment had a *p*-value of 0.068. Scheffe’s post hoc analysis indicated significant differences in lateral bowing and distal coronal alignment (*p* < 0.05). Specifically, group IV exhibited significantly greater lateral bowing than group I by 3.52° and group II by 2.41°. Additionally, for distal coronal alignment, group IV showed a significantly higher value compared to group I by 2.37° and group II by 2.05°. Linear regression analysis of bowing variables (R^2^ = 0.697, adjusted R^2^ = 0.677, *p* = 0.00) indicated that distal coronal alignment was not influenced by anterior bowing (*p* = 0.793), distal sagittal alignment (*p* = 0.178), or DOC (*p* = 0.178).

Regarding the age-related changes in coronal and sagittal FSB and alignments, an anterolateral bowing of the distal femur was observed, associated with a decrease in the DOC. Consequently, this condition resulted in a significant limitation of the potential space available for a straight femoral stem. The results of the correlation analysis on overall variables of FSB are outlined in Table 4. The distal sagittal alignment was significantly correlated with that of distal coronal alignment (correlation coefficient: 0.400, *p* = 0.001), lateral bowing (correlation coefficient: 0.326, *p* = 0.007), and DOC (correlation coefficient: −0.306, *p* = 0.011).

## 4. Discussion

The rapid advancement toward super-aged societies in certain Asian countries, including South Korea, indicates the swift progression of such counties toward this demographic transition. TKA in older adults has become a considerable economic burden on national healthcare systems, particularly in complex cases characterized by extensive social costs and functional impairment. Mechanical realignment and optimal component positioning are widely recognized as crucial for successful TKA outcomes. While computer-assisted surgery has rapidly advanced and is effectively utilized to achieve these objectives, it is essential to emphasize the surgeon’s creativity, which is rooted in a comprehensive understanding of knee anatomy, as a fundamental requirement. However, despite numerous studies on age-, bisphosphonate-, and ethnicity-related changes in hip geometry [13,14,15,16], there are few studies [17,18,19] on achieving better fit and optimal positioning of the implant. To the best of our knowledge, there are currently no existing reports on a clinical CT-based 3D modeling study of the Asian femur that assesses morphological features and implications for TKA using computational simulation at actual size.

In our study, we found several interesting and practical findings. Despite increased anterior bowing in the Asian ethnicity, the mean distal sagittal alignment was 2.66° (range, −1.24–7.35°). Considering that there was some difference between distal sagittal alignment and distal anterior cortex line, outliers are highly likely to occur in the case of osteotomy along the anterior cortical bone to prevent anterior notching. Furthermore, the mean coronal alignment of the distal femur was 6.40°, showing a relatively broad distribution. The empirical distal femoral valgus cut angle of 4–6° may lead to outliers when using conventional IM-guided rod techniques. Regarding the anterolateral bowing of the distal femur, the potential space for IM implantation, including the guided rod and femoral stem, may be significantly restricted. This restriction could potentially cause cortical abutment, particularly in the sagittal plane.

**Figure 4:**
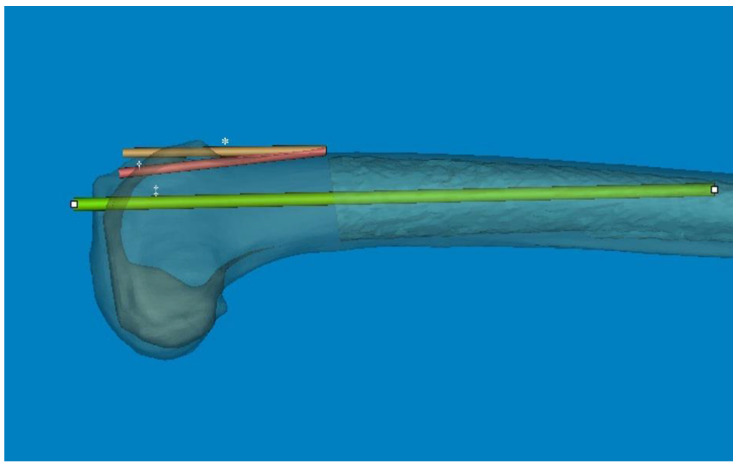
The angular difference between the anterior cortex line and distal centerline was significant. So, the distal metaphysis area was more flexed than the distal shaft. * The distal centerline is shifted to the anterior cortex. ^†^ Distal anterior cortex line. ^‡^ Distal centerline, extended from distal articular surface to the midshaft.

Accurate sagittal alignment of the femoral component in TKA is crucial for prosthesis longevity, improved function, and patient satisfaction. Navigation has been helpful in achieving an appropriate femoral sagittal alignment [20]. While the flexion of the femoral component was highly varied in conventionally aligned TKA [21], a recommended femoral sagittal alignment within 3° of flexion could be acquired in only 25% of the studied cases [22]. In our computational measurement method, we observed a difference between the distal sagittal alignment and the distal anterior cortex line (axis) by shifting the distal centerline anteriorly toward the anterior cortex. This adjustment was made in the osteotomy line to prevent anterior notching (Figure 4). Regarding these features of distal sagittal alignment, the femoral implantation along the distal femur bowing without anterior notching is anticipated to be an outlier, especially in patients older than 70 years. Chung et al. [23] and Seo et al. [24] found that the angular differences were on average 3.8° (range, −0.4–8.5°; SD, 1.7) and 2.4° (range, 0.4–4.2°; SD, 0.9) between the MA and sagittal anatomical axes of the distal femur. To obtain a true lateral radiograph of the entire femur, patients positioned their thighs on a digital flat detector at a diagonal angle, while the X-ray tube was accordingly tilted [23]. Hence, when comparing our method, it is important to note the potential for projection errors, especially since coronal bowing was not considered. The variable of the distal medullary axis closely resembled our distal sagittal alignment and distal anterior cortex; however, it did not precisely align with our distal cortical axis, which was situated along the osteotomy line to mitigate anterior notching. In addition, they described that the angular difference between the MA and distal anterior cortex was 3.0° on average (range, −1.0–8.5°; SD, 1.9) [23]. However, considering that the distal femoral anterior axis exhibited more flexion than the sagittal MA in our 3D femur model, the angular difference is significant. Therefore, it is likely that a new method to control the flexion angle of the component will be necessary, based on the distal sagittal alignment (Figure 4).

**Figure 5:**
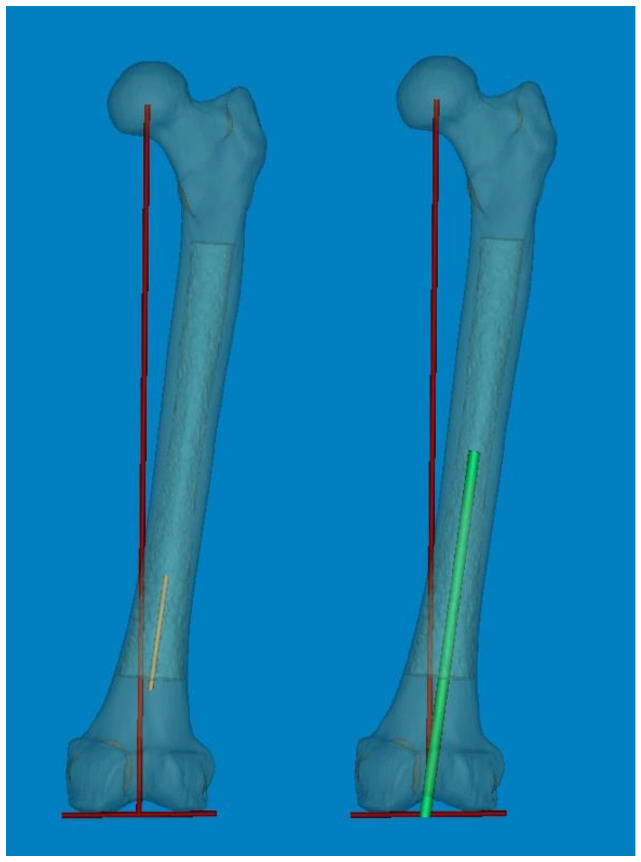
The individual distal femoral valgus cut (DFVC) angle could be anticipated by increasing the length of the distal centerline like an IM-guided rod.

Generally, the optimal coronal alignment has been determined to be within 3° of the MA [2,25]. The accuracy of femoral component placement using IM-guided rods depends on both patient-specific and surgeon-related factors. The selection of the optimal entry point and DFVC angle, considering the morphologic features of the femur, is critically important [26]. Although the DFVC angle should ideally be tailored to individual patient characteristics and preoperative deformities, it is commonly selected empirically within a range of 4° to 6°. This choice is based on the assumption that the angle between the femoral anatomical axis and the mechanical axis is approximately 5°. In our computational simulation study, the distal coronal alignment could be utilized to adjust the distal femoral valgus angle by increasing the distal centerline, similar to an IM-guided rod (Figure 5). The distal coronal alignment of all femurs was on average 6.40° (range, 1.98–11.17°; SD, 2.14) and showed a broad distribution. Due to coronal FSB, accurately drawing the femoral anatomical axis according to conventional definitions become challenging. Therefore, by considering FSB as an individual deformity, our method of distal coronal alignment, which was the angle between the MA and the distal straight centerline of the medullary canal, provides a practical approach for surgeons to examine FSB and estimate the best angle for DFVC without needing specialized software or equipment.

**Figure 6:**
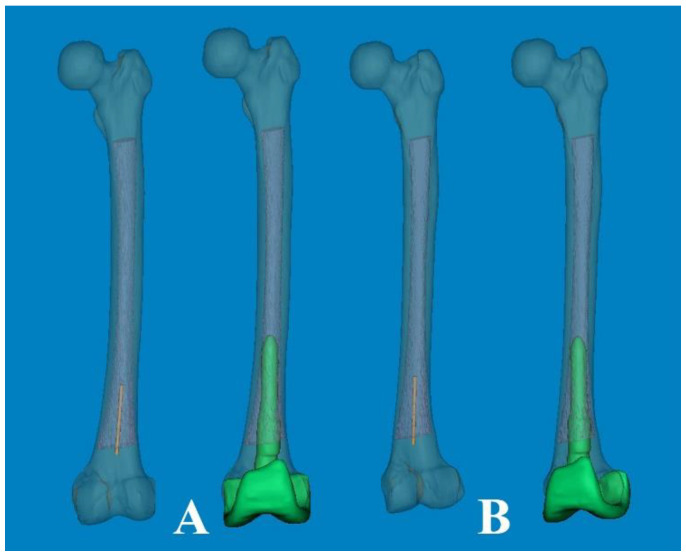
(**A**) Conventional AP radiographs show the lateral bowing of the femoral shaft. (**B**) Modified AP radiographs with approximately 15° of internal rotation show a more accurate medullary canal and intramedullary position of the femoral component in the coronal plane.

**Figure 7:**
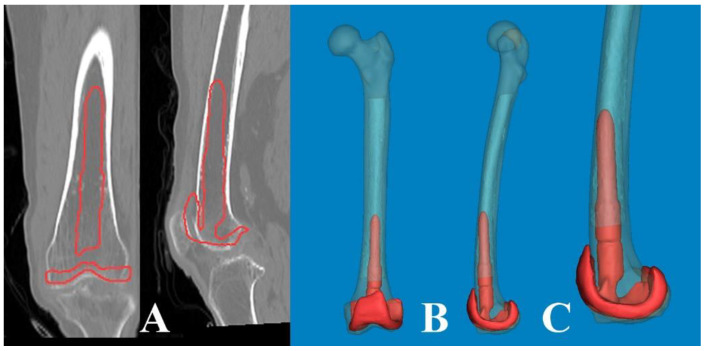
The lateral bowing of the proximal shaft was changed according to the CT scanning plane. (**A**) By scanning anterior to the midline of the head, the medullary canal seemed to be straight. (**B**) The CT scanning plane along the lag screw provides clear visualization of the lateral bowing. (**C**) The scanning plane posterior to the midline appears to be more bowed.

**Figure 8:**
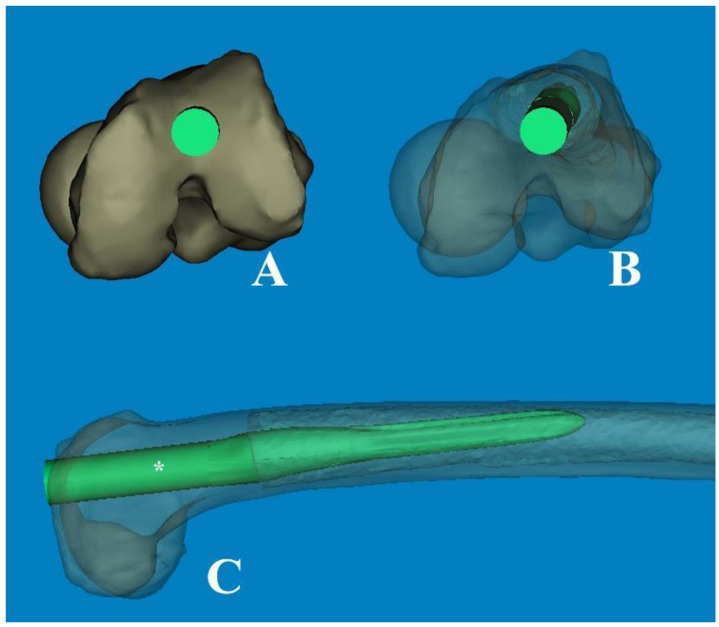
(**A**) The modified femoral stem, which changed from straight to curved (ROC of 4°), is inserted through the optimal entry point. (**B**) The transparent cephalad view showed the morphologic features of the medullary canal and the intramedullary position of the stem, which was rotated anterolaterally. (**C**) The cortical abutment between the anterior cortex and the implant could be avoided by using the curved stem. * The length of 180 mm.

As a part of the preoperative planning for TKA, it is crucial to consider the morphological characteristics of the medullary canal, particularly in revision surgery. This consideration is important because the femoral stem must be inserted deeply enough to achieve endocortical contact in the distal femur. As widely understood from atypical femur fractures, FSB increases in the anterolateral direction with aging. Thus, the effective medullary space for a straight femoral stem is limited. In our computational simulation, we utilized a 3D rendering program (Mimics^®^ software, Version 21.0) that allowed for free 360° rotation with magnification in any plane. This enabled us to implant the femoral component of TKA virtually in the optimal or intentional position. Interestingly, regarding anterolateral FSB in Asian geriatric femur, it was observed that AP radiographs with approximately 15° internal rotation (modified femur AP radiographs) reduced the projection error and enabled a more accurate medullary space measurement in the coronal plane (Figure 6). Furthermore, our findings demonstrate that cortical abutment can occur in the sagittal plane, despite limited space in the coronal plane (Figure 7). To overcome the discrepancy between the implant and the curvature of the distal femoral shaft, especially concerning the unique characteristics of the geriatric Asian femur, femoral stem design could be adapted to incorporate a radius of curve. Through computational simulation of the modified femoral stem (radius of curve of 4°), the cortical abutment between the anterior cortex and the femoral stem can be avoided by rotating the implant anterolaterally (Figure 8).

This study has some limitations. Firstly, because all the measurements were derived from normal femurs, our findings may be more descriptive compared to those of clinical studies. Secondly, since all the measurements were manually conducted using Mimics^®^ software, there is a possibility of both intra- and inter-observer errors. Thirdly, with a relatively small sample size consisting of only 68 femurs of Asian ethnicity, the generalizability of the results may be constrained. Fourthly, the resolution of CT scanning could affect the accuracy of measurements.

Despite these limitations, this study represents the first attempt to synchronously measure sagittal and coronal bowing using a 3D computational simulation method at actual size. Our significant findings on FSB are valuable in elucidating the anatomy of the geriatric Asian femur and its implications for femoral component alignment. This knowledge can aid in modifying implant designs and mitigating concerns related to cortical abutment concerns.

## 5. Conclusions

The Asian femur exhibited a mean lateral FSB of 3.7° and an anterior FSB of 11.8°, with variations observed in three types: coronal, sagittal, and both. With aging, anterolateral FSB increases, leading to potential cortical abutment in the sagittal plane during TKA despite some space in the coronal plane. Modified AP radiographs with approximately 15° of internal rotation can provide more accurate measurements of medullary space and distal shaft bowing in the coronal plane. When performing TKA in femurs with FSB as an individual deformity, distal coronal alignment can be used as the DFVC angle. Considering the difference between distal sagittal alignment and distal anterior cortical axis, caution is necessary to avoid outliers, especially in cases of osteotomy along the anterior cortical bone, to prevent anterior notching.

## Figures and Tables

**Table 1:** Overall characteristics and comparison of Asian femur based on sex.

	Total (68)	Male (35)	Female (33)	*p*-Value
Age (years)	69.1 (range, 33–93, SD: 13.97)	69.1 (range, 33–93, SD 13.97)	69.1 (range, 33–93, SD 13.97)	0.059
FSB_no (19)	19	14	5	
FSB_coronal ^1^ (7)	22	1	6	0.006 (χ^2^ = 7.624 *)
FSB_sagittal ^2^ (27)	42	15	12	0.419
FSB_both ^3^ (15)	15	5	10	0.020 (χ^2^ = 9.784 *)
Lateral bowing (°) ^4^	3.71 (range, 0.0–10.1, SD: 2.71)	2.37 (range, 0.0–7.5, SD: 2.05)	5.14 (range, 0.5–10.1, SD: 2.63)	0.000
Anterior bowing (°) ^5^	11.82 (range, 4.5–18.5, SD: 2.97)	11.17 (range, 5.7–15.4, SD: 2.62)	12.50 (range, 4.5–18.5, SD: 3.20)	0.065
Coronal alignment (°) ^6^	6.40 (range, 1.98–11.17, SD: 2.14)	5.61 (range, 1.98–9.37, SD: 1.84)	7.24 (range, 2.15–11.17, SD: 2.16)	0.001
Sagittal alignment (°) ^7^	2.66 (range, −1.24–7.35, SD: 2.06).	2.16 (range, −1.24–5.31, SD: 1.60)	3.18 (range, −1.24–7.35, SD: 2.06).	0.044
Diameter_BM ^5^ (mm) ^8^	1501.6 (range, 979.6–2515.8, SD: 317.70)	1567.1 (range, 1139.5–2515.8, SD: 339.41)	1432.3 (range, 979.6–2083.9, SD: 281.56)	0.080

FSB, femoral shaft bowing; BM. * Comparison of continuous data (mean ± SD) was performed using the independent samples t-test. For categorical data, Person’s chi-square tests were applied. ^1^ FSB >5° in the coronal plane was defined as lateral bowing (+). ^2^ FSB >11° in the sagittal plane was defined as anterior bowing (+). ^3^ Lateral bowing (+) and anterior bowing (+). ^4^ Diaphysal bowing in the coronal plane, the acute angle formed by the meeting of the proximal and distal straight line. ^5^ Diaphysal bowing in the sagittal plane, the acute angle formed by the meeting of the proximal and distal straight line. ^6^ Mechanical axis line in the coronal plane, the acute angle formed by the meeting of the mechanical axis and distal straight line. ^7^ Mechanical axis line in the sagittal plane, the acute angle formed by the meeting of the mechanical axis and distal straight line. ^8^ Diameter of the curve of the medullary canal along the posterior border.

**Table 2:** Overall characteristics and comparison of Asian femur based on an age cutoff of 70 years.

	Younger Group (28)	Older Group (40)	*p*-Value
FSB_No	15	4	
FSB_coronal	3	19	0.001 (χ^2^ = 10.184)
FSB_sagittal	13	29	0.027 (χ^2^ = 4.741)
FSB_both	3	12	0.000 (χ^2^ = 19.059)
Lateral bowing (°)	2.02 (range, 0.0–5.3, SD: 1.59)	4.90 (range, 0.3–10.1, SD: 2.72)	0.000
Anterior bowing (°)	10.60 (range, 5.7–15.4, SD: 2.50)	12.67 (range, 4.5–18.5, SD: 3.01)	0.003
Coronal alignment (°)	5.22 (range, 1.98–8.61, SD: 5.22)	7.23 (range, 2.15–11.17, SD: 2.15)	0.000
Sagittal alignment (°)	1.90 (range, −1.24–5.64, SD: 1.66)	3.19 (range, −0.62–7.35, SD: 2.17).	0.007
Diameter_BM (mm)	1601.8 (range, 1015.2–2515.8, SD: 365.0)	1431.6 (range, 979.6–2083.9, SD: 262.58)	0.040

FSB, femoral shaft bowing; BM.

**Table 3:** Overall characteristics and comparison of Asian femur based on the age group *.

	Group I (14)	Group II (14)	Group III (21)	Group IV (19)	*p*-Value
FSB_no ^†^	8	7	2	2	
FSB_coronal	1	2	10	9	0.016 (χ^2^ = 10.347)
FSB_sagittal	6	7	15	14	0.178
FSB_both	1	2	6	6	0.021 (χ^2^ = 19.498)
Lateral bowing (°)	1.47 ± 1.40	2.57 ± 1.62	4.82 ± 3.02	4.98 ± 2.43	0.001
Anterior bowing (°)	10.34 ± 2.53	10.84 ± 2.53	12.49 ± 2.81	12.86 ± 3.29	0.035
Coronal alignment (°)	5.10 ± 1.38	5.39 ± 1.66	7.04 ± 2.34	7.43 ± 1.97	0.001
Sagittal alignment (°)	1.59 ± 1.51	2.21 ± 1.81	3.20 ± 2.28	3.18 ± 2.10	0.068
Diameter_BM (mm)	1713.3 ± 399.50	1490.3 ± 300.49	1416.7 ± 258.451	1448.0 ± 273.22	0.037

FSB, femoral shaft bowing; BM, curve of the medullary canal along the posterior border. four groups, group I: under 60 years old, group II: 60 to 69 years old, group III: 70 to 79 years old, group IV: over 80 years old. * ANOVA was used to compare continuous data among four groups, and Scheffe’s post hoc test was used for multiple comparisons. ^†^ No Femoral shaft bowing.

**Table 4:** Overall correlation of FSB.

	Lateral Bowing	Anterior Bowing	Coronal Alignment	Sagittal Alignment	Diameter of BM
Lateral bowing	1				
Anterior bowing	0.288 *	1			
Coronal alignment	0.816 **	0.303 *	1		
Sagittal alignment	0.326 *	0.758 **	0.400 **	1	
Diameter of BM	0.254 *	0.604 **	0.143	0.306 *	1

FSB, femoral shaft bowing; BM. Pearson’s correlation coefficients were used for correlation analysis of normally distributed data.* Pearson’s correlation is significant at the 0.05 level (two-tailed). ** Pearson’s correlation is significant at the 0.01 level (two-tailed).

## Data Availability

The data that support the findings of this study are available from the corresponding author, Dong-Geun Kang, upon reasonable request.
